# Incorporation of Conductive Materials into Hydrogels for Tissue Engineering Applications

**DOI:** 10.3390/polym10101078

**Published:** 2018-09-28

**Authors:** Ji Hong Min, Madhumita Patel, Won-Gun Koh

**Affiliations:** 1 Department of Chemical and Biomolecular Engineering, Yonsei University, Seoul 03722, Korea; jh_min@yonsei.ac.kr (J.H.M.); madhurk29@gmail.com (M.P.); 2 Active Polymer Center for Pattern Integration (APCPI), Yonsei-ro 50, Seoul 03722, Korea

**Keywords:** conductive hydrogel, tissue engineering, biomaterials, physical and electrical properties

## Abstract

In the field of tissue engineering, conductive hydrogels have been the most effective biomaterials to mimic the biological and electrical properties of tissues in the human body. The main advantages of conductive hydrogels include not only their physical properties but also their adequate electrical properties, which provide electrical signals to cells efficiently. However, when introducing a conductive material into a non-conductive hydrogel, a conflicting relationship between the electrical and mechanical properties may develop. This review examines the strengths and weaknesses of the generation of conductive hydrogels using various conductive materials such as metal nanoparticles, carbons, and conductive polymers. The fabrication method of blending, coating, and in situ polymerization is also added. Furthermore, the applications of conductive hydrogel in cardiac tissue engineering, nerve tissue engineering, and bone tissue engineering and skin regeneration are discussed in detail.

## 1. Introduction

A hydrogel, which can stimulate the function of native tissues, has been an increasingly essential issue in the field of tissue engineering resulting from aging, injuries, or diseases [1,2]. It can provide a 3D hydrated polymeric network that can be synthesized in various shapes and sizes because of its unique physical properties. Mimicking a complex tissue structure and providing an essential cellular microenvironment are essential elements that need to be considered to manage the formation of functional tissue in a fabricated hydrogel. However, cell guidance to the differentiated cells is still a challenge.

Metal nanoparticles and carbon-based materials are widely reported in tissue engineering applications due to their electrical properties [3,4] but their long-term cytotoxicity and homogeneous distribution have restricted their wider use. Conductive hydrogel is one of the most effective materials with which to replicate the electrical and biological characteristics of biological tissues that require the most conductivity [2,5,6]. The combination of a soft hydrogel and a conductive polymer is known as a conductive hydrogel. Conductive materials such as polypyrrole (PPY), polyaniline (PANi), polythiophene (PT), poly(3,4-ethylene dioxythiophene) PEDOT, and poly(p-phenylene vinylene) PPV are widely used biomaterials. These materials enhance cell adhesion, proliferation, and differentiation with or without electrical stimulation [7,8].

The advantage of a conductive hydrogel is that it can provide both physical and electrical properties, in which the former is the unique property of the hydrogel and the latter is the conductivity performed by the conductive materials [2,9]. There have been many studies of designed biomaterials with controlled electrical properties that would be useful for promoting the formation of functional tissues [10,11]. To provide a cell-effective conductive environment, conductive hydrogels have been synthesized via various techniques and with conductive materials that either obtain biocompatibility or effectively provide an electrical cue to cells for restoring the functions of cellular tissues and satisfying the demanding needs of biomedical applications.

In this review, we first focus on detailed introductions of the types of conductive hydrogels; in particular, we focus on emerging trends in conductive materials such as metal nanoparticles, conductive polymers, and carbons. Details are provided of the methods for synthesizing conductive hydrogels based on a blending process, in situ process, and coating process. We also address biomedical applications in the cardiac and neuronal fields, which have been actively studied in the field of tissue engineering using conductive hydrogels. Then, we discuss the future perspective of conductive hydrogels in the field of tissue engineering.

## 2. Types of Conductive Hydrogels

The conductive hydrogel can implement a variety of fabrication systems depending on the type of materials or fabrication methods.

### 2.1. Materials

#### 2.1.1. Metal Nanoparticles

**Figure 1:**
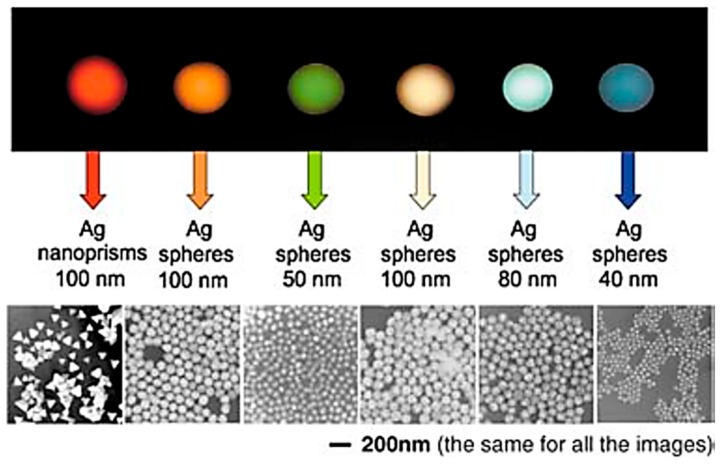
Nanoparticles behave differently depending on the size and shape of the material. This figure shows the difference between the Rayleigh light-scattering properties of silver nanoparticles (reproduced from [12] with permission, copyright Wiley, 2005).

Metal nanoparticles are nanometer-sized ultrafine particles and behave differently depending on the type, shape, and size of the material (Figure 1) [12,13]. The optical characteristics, as well as the conductivity of metal nanoparticles, are dependent on the particle size [14]. By functionalizing nanoparticle surfaces, the interaction between polymers and nanoparticles can be strengthened [15,16,17]. Various types of metal nanoparticles have been used in the production of nanocomposite hydrogels in the field of biomaterials including gold [18], silver [19], and other noble metal nanoparticles, while metal oxide nanoparticles such as iron oxide [20] and zirconia [21] have also been used. Since metal and metal oxide nanoparticles possess the desired electrical conductivity, magnetic properties, and antibacterial properties, nanocomposite hydrogels that contain metal or metal oxide nanoparticles are widely used in conductive scaffolds, electronic switches, actuators, and sensors [22,23,24,25] (Table 1).

**Figure 2:**
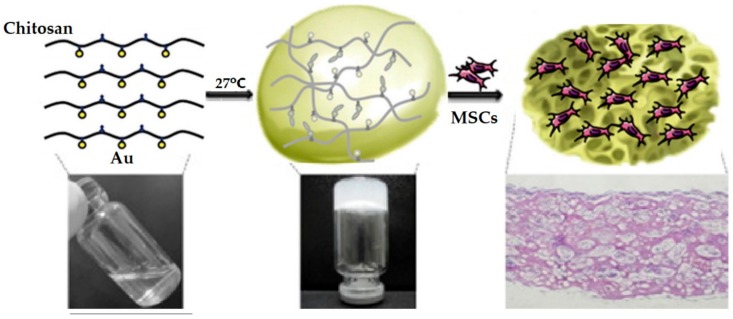
Thermosensitive conducive hydrogel by combining AuNPs with chitosan. The potential of AuNPs as a material of conductive hydrogel was confirmed (reproduced from [34] with permission, copyright Elsevier, 2016).

Gold nanoparticles (AuNPs) are one of the most essential metal nanoparticles actively used in biomedical fields. Methods for synthesizing AuNPs generally include nanoscale lithography and chemical, electrochemical, photochemical, and thermal reduction techniques [26], and have led to the creation of various shapes and sizes of AuNPs. They have been utilized in various visualization and bioimaging techniques [27], photothermal therapy for targeting the injury of tumor tissue sites [28,29,30,31], antigen detection, and immunostaining research used in radioactive labeling [32]. In addition, gold metals can provide electrical conductivity. Conductive hydrogels can be developed so that they can provide both hydrogels and additional attributes of AuNPs. Although AuNPs are weak optical signals, they have long-term cytotoxicity [33]. Baei et al. synthesized a thermosensitive conductive hydrogel by combining AuNPs with chitosan (Figure 2) [34]. The gelation and conductivity of the hydrogel were controlled by the concentration of the AuNPs and supported the metabolism, viability, migration, and proliferation of myocardial cells. Chitosan was fabricated to thermoresponsive hydrogel, through the simple addition of polyol salts such as glycerol phosphate, and kept at 37 °C until gelation.

Silver nanoparticles (AgNPs) have also commonly been used in biomedical applications because of their inherent characteristics of unique optical, electronic, and antibacterial properties. Synthesizing techniques for AgNPs include laser cutting, gamma irradiation, electron irradiation, chemical reduction, photochemical methods, microwave treatment, and biological synthesis methods [35]. Based on these techniques, the adjustment of size and the agglomeration of AgNPs can control antimicrobial activity [36,37,38,39,40]. Silver nanostructures are used for imaging and bio-diagnostics due to their optical and photo-thermal properties [41,42]. AgNPs are promising materials that can be utilized in the production of conductive hydrogels and provide unique factors including strong antibacterial effects, optical properties, and conductivity. The excessive antibacterial effect or the excessive agglomeration of silver nanoparticles sometimes lead to cell death. Nevertheless, it is expected that AgNPs can be used in conductive hydrogels to control conductivity and optical/antibacterial properties.

Platinum nanoparticles (PtNPs) are promising versatile metal nanoparticles that have been applied in various research applications in recent years. Various synthesis methods have been devised for PtNPs, including chemical reduction using chemical solutions and physical synthesis using electron beam evaporation [43]. PtNPs have been used as catalysts, biosensors, and in many other biomedical applications because of their unique catalytic and optical properties. In particular, detection using PtNPs showed excellent catalytic properties and has been used for the electrochemical analysis of living bodies [44,45]. In addition, research results have reported that PtNPs can be used as biocatalysts through various shapes of PtNPs, such as nanotubes and nanofibers [46]. Despite successful results, PtNPs have limited applicability in the field of biomedical research. However, conductive hydrogels with PtNPs have been expected and studied as a bioreactor because of the catalytic property of PtNPs.

Metal oxides are known to exhibit interesting nanomorphisms or functional biocompatibility, non-toxicity, and catalysis. These materials have high electron transfer kinetics or strong adsorption ability that can provide an environment that is suitable for the immobilization of biomolecules and can impart improved electron transfer and biosensing characteristics. Iron oxide nanoparticles have been used in many in vivo applications including the enhancement of magnetic resonance imaging contrast and treatment, tissue repair, immunoassay, fluid decontamination, and cell sorting [47,48]. Zinc oxide nanoparticles are metal oxides with a wide range of applications and possess unique optical, chemical sensing, antibacterial [49,50], electrical conductivity, and piezoelectric properties [51]. In addition, research results have shown that TiO_2_ [52] and ZrO_2_ [53] nanoparticles enhance the strength and conductivity of supported substrates.

#### 2.1.2. Conductive Polymers

**Figure 3:**
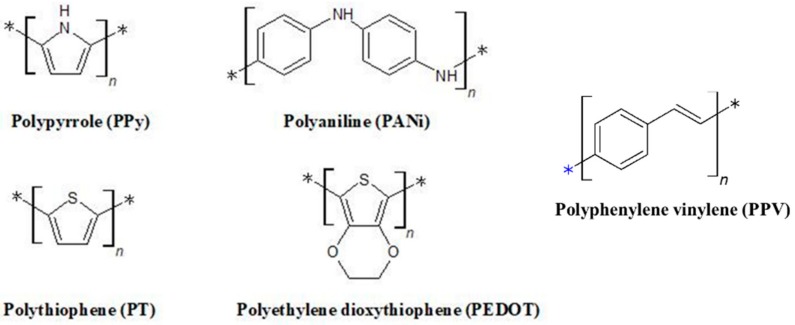
Chemical structures of various conductive polymers.

Conductive polymer (CP) is an organic an electronically conjugated polymer material loosely fixed on a backbone with electro-optic properties similar to those of metals [54]. Since pi-electrons move freely, they can form electrical pathways of mobility charge carriers [55,56]. The usage of conducting polymers allows a hydrogel to provide electrical stimulation locally and enhance the physical properties of the hydrogel as a template to accurately control the extent and duration of external stimulation [57,58,59]. Conductive materials such as polypyrrole (PPy), polyaniline (PANi), polythiophene (PT), and poly(3,4-ethylene dioxythiophene) (PEDOT) have been widely used in conductive hydrogels (Figure 3, Table 2).

**Figure 4:**
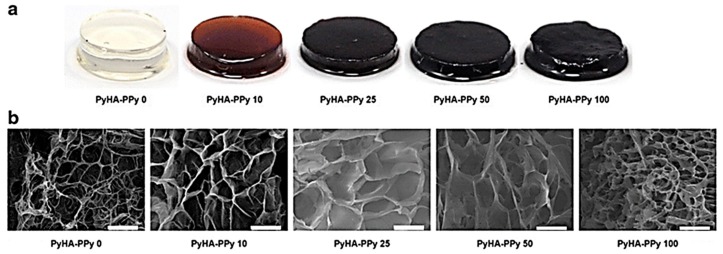
PPy/hyaluronic acid hydrogels; (**a**) various PyHA-PPy hydrogels and (**b**) SEM images of PyHA-PPy hydrogels. Scale bars are 50 μm. (reproduced from [71] under open access license).

As the most studied conductive polymer, PPy has been synthesized by chemical oxidation using a radical initiator with an appropriate electrolyte solution [60,61] or by electrochemical oxidation of pyrrole with an electrolyte solution on a platinum-coated electrode [62]. PPy has been reported to promote focal adhesion and the growth of various cell types associated with endothelial cells [63,64], neurons, supporting cells (DRG) [65,66,67], and rat pheochromocytoma (PC12) cells [67,68,69,70]. Yang et al. devised conductive hydrogels of hyaluronic acid and PPy that enhanced mechanical and conductive properties [71] (Figure 4). In this study, PPy/hyaluronic acid hydrogels were 5-fold of Young’s modulus compared to uncoated hyaluronic acid hydrogels and had 7.3 mS·cm^−1^ of conductivity. However, the unreformed and straightforward form of PPy can be synthesized to have an additional small biological anion (Cl^−^) as a dopant to confer additional biological properties or to improve the stability of PPy. It can be innovated to support growth of various cell types and to encourage specific aspects of wound healing by simply changing the dopant [72,73,74]. Furthermore, it is essential to consider controlling the mechanical properties. Since unchanged PPy, like most other CPs, is crystalline, fragile, and susceptible to irreversible oxidation [75], there is no ideal candidate for tissue support materials. Therefore, to overcome such disadvantages, the development of dopants and PPy analogs is continuously being researched [76].

**Figure 5:**
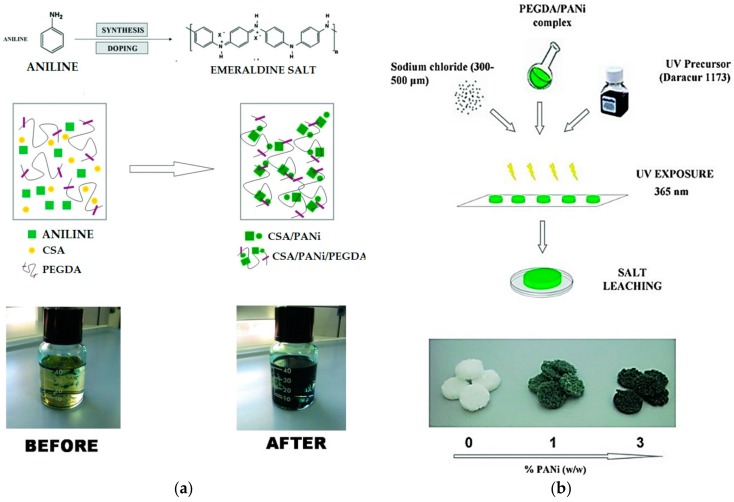
(**a**) In situ CSA-PANi synthesis in PEGDA solution and (**b**) preparation of PANi/PEGDA microporous hydrogels (reproduced from [80] with permission, copyright Wiley, 2012).

Another frequently used CP is PANi, which is a substance polymerized chemically or electrochemically with monomeric aniline. Several strategies have been proposed on the development of PANi with excellent cardiac and PC12 cell compatibility [77], conductivity, and mechanical properties. As a result of studying the in vivo response of PANi in various oxidation state implants, it was confirmed that severe inflammation did not occur in the implant site in general [78]. Although several studies report that PANi is not overtly cytotoxic, it needs to be better modified for better cell adhesion and proliferation [79]. For these reasons, various methods have attempted to physically fabricate hydrogel with the desired electrical properties of the material and PANi [80,81]. For example, PANi-PEG conductive hydrogel was prepared by the precipitation of PANi in a polyethylene glycol diacrylate (PEGDA) solution; then, crosslinking of polymer chains occurs under UV irradiation (Figure 5) [80]. The hybrid material improved conductivity with its hydrophilic nature and showed that optimization of 3 wt.% PANi improved the biological reaction of PC12 and human mesenchymal stem cells (hMSCs) in an in vivo study. In another study, PANi was grafted to the gelatin backbone by genipin at body temperature. The conductivity of the hydrogel increased with the increased content of PANi and exhibited non-cytotoxicity with BMSC and C2C12 cells [81]. Dong et al. synthesized chitosan-g-PANi (QCSP) hydrogel, in which PANi was grafted to the QCS backbone. The hydrogel showed self-healing properties, antibacterial properties, and similar conductivity to the native cardiac tissue (~10^−3^ S cm^−1^). In addition, the hydrogel showed good biocompatibility with the C2C12 myoblast cells and H9C2 cardiac cells [82].

For the purpose of tissue engineering, various CPs including PT and new CP were sought in addition to the most studied PPy and PANi for conductive hydrogels. PT is synthesized with various cross-coupling reactions using transition metal, nickel and palladium catalysts, oxidative polymerization, electrochemical polymerization, and biocatalyzed polymerization. PTs can easily acquire various functions by the organic reaction of substituted thiophene monomers, and new properties can be obtained through the polymerization of these functionalized monomers [83]. Although the shortcomings of conductive stability and mechanical integrity have been a problem for long-term performance deficiencies, the functionalized PT of the optimized structure can be used to alleviate the problems of existing materials.

**Figure 6:**
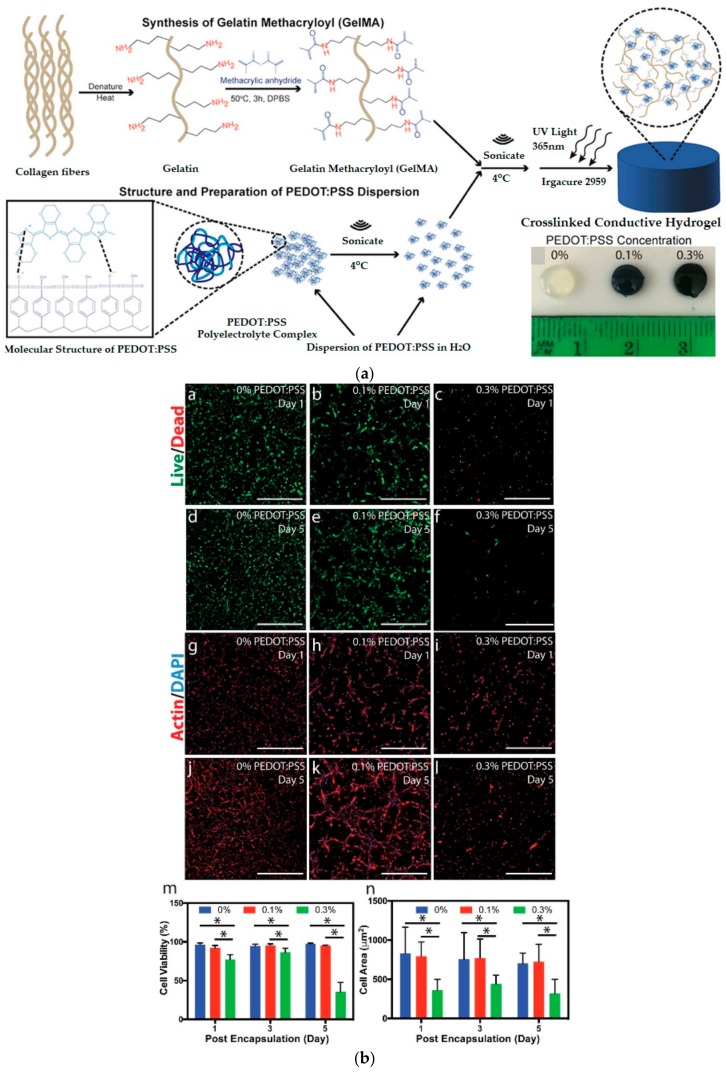
Study of GelMA/PEDOT:PSS hydrogels (**a**) scheme of GelMA/PEDOT:PSS hydrogel synthesis and (**b**) representative LIVE/DEAD images and quantification of cell spreading from C1C12 cells encapsulated in different amounts of PEDOT:PSS (reproduced from [90] with permission, copyright American Chemical Society, 2018).

PEDOT is utilized in various studies, because it has biocompatibility characteristics similar to those of polythiophene derivatives and melanin, which are natural biological substances [84,85,86]. Another advantage of PEDOT is that its monomer is hydrophilic, thus enabling it to be soluble in water and making its composition easy-to-tailor by blending with different materials in the synthetic aqueous system of other polymers [87,88]. In general, PEDOT has been doped into poly styrene sulfonate (PSS) to obtain excellent film-forming ability and hydrophilic polyelectrolyte system [89]. Spencer et al. prepared composite conductive hydrogels from PEDOT-PSS dispersed within photo-crosslinkable gelatin methacryloyl (GelMA) hydrogels (Figure 6) [90]. The doped PEDOT-PSS adjusts the band gap to improve conductivity and provide excellent stability in the doping state [91]. In addition, the advantages of PEDOT: PSS film are its compatibility and stability with most organic solvents during the manufacturing process [92].

PPV is a conductive material that can be processed into a highly ordered crystalline film. Well-doped PPV is synthesized as a conductive polymer with appropriate conductivity, which has nonlinear optical properties, electroluminescence, and high electric conductivity [93]. It is insoluble in water, but the precursor can become soluble and react in aqueous solution. Most PPV-based conductive hydrogels can change their density depending on the strength and conductivity of the electric field, so they have been often used as materials for drug delivery and release systems [94,95].

#### 2.1.3. Carbons

Graphene and carbon nanotubes (CNTs) applied as a conductive material of a biomatrix can enhance cell attachment and proliferation (Table 3). Graphene is a single layer mineral graphite that has variety of physical and chemical properties, which include superconduction, high surface area, excellent thermal conductivity, and high mechanical strength [95,96,97].

Graphene can be mass-produced by decomposing SiC wafers under graphene oxide (GO) chemistry, mechanical exfoliation, chemical vapor deposition, and liquid-phase exfoliation [94]. In the laboratory, although the yield is low, highly pyrolyzed graphite is repeatedly peeled off graphite to produce a graphene sheet [98]. Mechanical strength is one of the several advantages of using graphene and can be changed by adjusting the graphene concentration. Therefore, the preparation of hydrogel-containing graphene has been applied to various fields including energy storage [99], catalysts [100], and sensors [101]. Lee et al. showed that graphene film improved MSC proliferation and differentiation when compared to a polydimethylsiloxane (PDMS) film. The graphene film acts as a reserve platform for bone formation inducers and advances the growth of MSCs in the osteogenic lineage because of their strong non-covalent binding [102]. However, adjacent graphene sheets can interfere with applications [103] because of their serious aggregation owing to pi–pi interactions and cytotoxicity. Nevertheless, they are expected to be used in the production of conductive hydrogels through graphene surface modification, mixing with other materials, and hydrogel encapsulation to provide excellent conductivity.

GO, a representative oxide of graphene, is a mixture of sp2 and sp3 hybridized carbon atoms with a thin layer of graphite covalently attached to oxygen-containing functional groups [104]. Functional groups that consist of oxygen have the advantage of being easily dispersed in water and are capable of interacting with different inorganic and organic materials [105]. Several studies have confirmed that synthesized GO shows excellent biocompatibility, cell adhesion, and proliferation [106,107]. However, depending on the ambient humidity and proportion of oxide, the conductivity and physical properties of the GO can be restricted. To overcome these shortcomings, researchers have adopted the reduced form of GO (rGO) to partially recover the physical and electrical properties. rGO is superior to GO in conductivity and biocompatibility in the process of detecting enzyme-based reactions [108]. Although GO and rGO are very likely to be utilized in the field of tissue engineering as the main material adopted for synthesizing the conductive hydrogel for specific biocompatibility and conductivity, the relatively low conductivity and physical properties of GO and rGO can be a challenge in practical applications.

CNTs are cylindrical carbon tubes with nanometer diameters with a large aspect ratio. CNTs are generally manufactured by laser cutting, arc discharge, or chemical vapor deposition. Nitric acid-containing oxidants are used to remove catalysts in the refined process of CNT, which can regulate the chemical composition of CNT surfaces by making carboxylic acid groups at the terminal CNT end.

**Figure 7:**
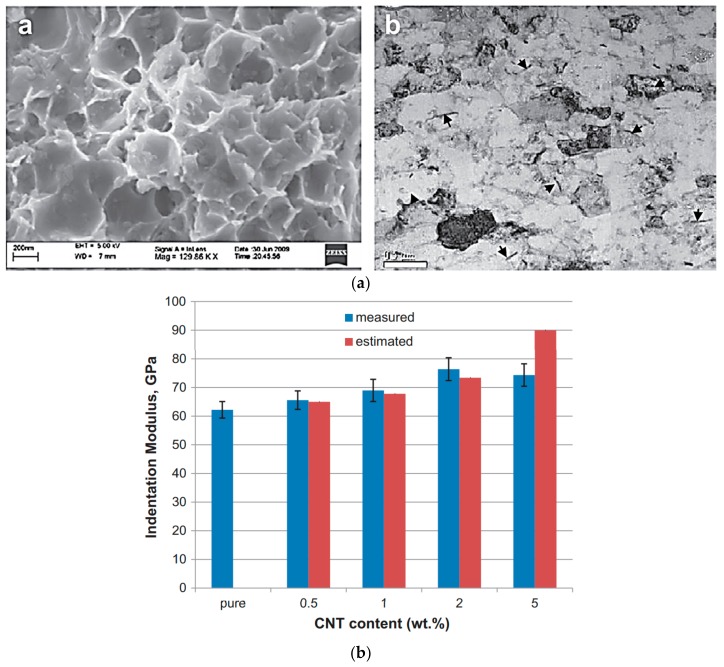
CNTs are suitable for strength reinforcement. (**a**) SEM image of surface of an Al–2 wt.% CNT and TEM image of an Al–2 wt.% CNT showing dispersed CNTs (indicated by arrows) within the Al matrix. (**b**) Effect of CNT content and estimated modulus values in the indentation modulus of investigated composites (reproduced from [112] with permission, copyright Elsevier, 2010).

CNTs are commonly utilized in biomedical applications (Figure 7) because of their high aspect ratio, low density, and electrical and physical properties [109,110,111,112,113,114]. Zhang et al. investigated the interaction between cells and modified multi-walled carbon nanotubes (MWCNTs) for biomedical applications [115]. In this study, the cell viability of human osteoblast MG-63 cells was increased by up to 67.23%. According to the results of several in vitro studies, however, CNTs can have cytotoxicity because of their inducement of oxidative stress [116] and their structure [117]. It has been reported that HeLa cells treated with functionalized single-walled carbon nanotubes (SWCNTs) and MWCNTs reduced the number of cells by 50% [118]. Nonetheless, approaches to mitigating toxicity have been discussed to exploit the advantages of CNTs. CNTs are still a promising material for producing conductive hydrogels, because they increase their strength and conductivity.

#### 2.1.4. Hybrid Materials

The conducting polymer is used, together with another polymer, to increase conductivity and mechanical strength. The hybrid materials improve conductivity more than a single conductive material. For example, it has been confirmed that a specific composition of CNT:graphene hybrid material has a higher conductivity than a 100% CNT or graphene material [119].

**Figure 8:**
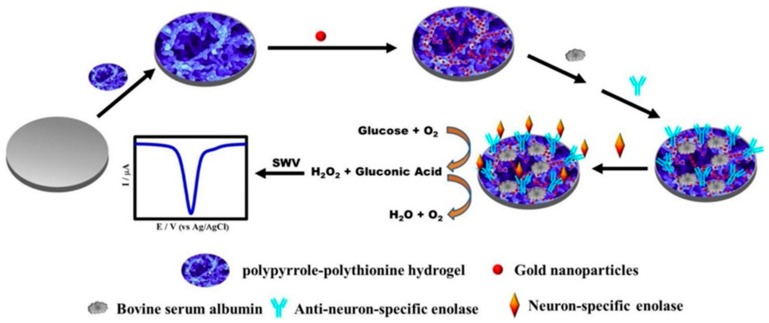
Schematic illustration of synthesized PPy-PT-Au with multifunctional conductive hydrogel for detection of tumor markers (reproduced from [120] with permission, copyright Elsevier, 2018).

Wang et al. synthesized PPy-PT-Au with multifunctional conductive hydrogels in glucose oxidase for the high sensitivity detection of tumor markers (Figure 8) [120]. In this experiment, a label-free amperometric immunoassay for neuron-specific enolase (NSE) was identified by binding to a hydrogel and resulted in a high detection limit ranging from 100 to 1 pg·mL^−1^. Li et al. synthesized a cylindrical Au/graphene hydrogel under hydrothermal conditions. The self-assembly catalyst reduced 4-nitrophenol (4-NP) to 4-aminophenol (4-AP) that is about 90 times higher than the AuNP sponge type and 14 times higher than the polymer-supported Au nanoparticles catalyst [121]. It promoted electron absorption by 4-NP molecules through the high adsorption power of graphene 4-NP and electron transfer of graphene to AuNPs.

To provide both conductivity and biocompatibility, research has been conducted to hybridize multiple substances and to confirm their effects [122,123]. A CNT-CP composite material can impart conductivity and show superior synergistic conductivity compared with CP and CNT, according to the results of a recent research for developing a conductive hydrogel [124]. Nevertheless, these materials have been widely used because of the high charge characteristics of CP or CNTs [125]. Conductive hydrogels composed of two or more conductive materials are expected to open the new possibilities in the tissue engineering field.

### 2.2. Synthesis Process

Many researches have studied the combination of hydrogels with conductive materials. To provide conductivity to a hydrogel, methods such as agitation of the synthesized conductive materials in the hydrogel-forming process, synthesis in situ within the hydrogel, and coating of the surface of the hydrogel have been performed [126] (Table 4). Since the conducting environment provided to each cell differs depending on the method of introduction of the conductivity of the hydrogel [58], it is essential to select a method that is suitable for each cell and application.

#### 2.2.1. Blending Process

Many experiments have been carried out on conductive hydrogels in the form of conductive components dispersed in a hydrogel. Generally, pre-fabricated conductive materials are added to a polymer solution before the formation of a hydrogel. It is essential that the conductive component achieves homogeneous mixing so that the conductive path is generated in a nonconductive hydrogel network. It is important that the metallic particles incorporated in hydrogels form interconnecting pathways of particles for electron transfer without compromising the physical properties of the hydrogel [127].

For example, metallic materials including micro/nanoparticles and wires made of a metal, such as gold or silver, are introduced inside the hydrogels to impart electrical properties. Although it is difficult for a network of nanowires to control uniform distribution [128,129,130,131,132,133,134], conductive hydrogels with nanowires can be fabricated for a wide range of tissue engineering fields, such as pressure sensors, biosensors, and electrophysiological catheters [135,136,137].

**Figure 9:**
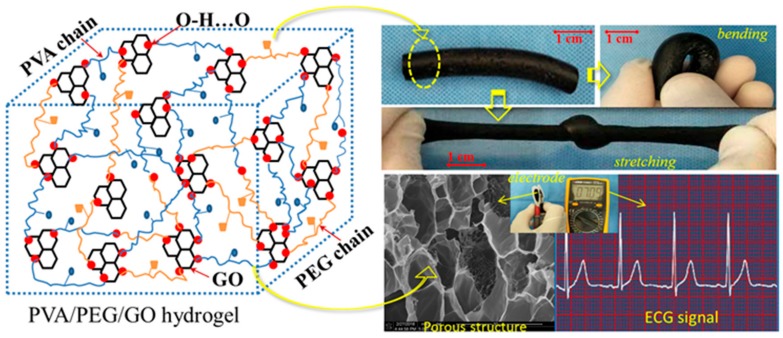
Polyvinyl alcohol/PEG/GO hydrogels blending with GO using the freezing thawing method (reproduced from [138] under open access license).

Xiao et al. synthesized conductive hydrogels with high mechanical strength and electrical conductivity using polyvinyl alcohol, polyethyleneglycol (PEG), and GO nanoparticles (Figure 9) [138]. During the freezing process of the GO solution in the mixed solution, crosslinking occurred for high mechanical performance. After the 3D network structure was successfully synthesized, the polymer network formed by the dense hydrogen bonds showed high strength and elasticity.

Meanwhile, in the process of synthesizing a conductive hydrogel based on a blending method, there are production difficulties with the heterogeneous aggregation of the conductive material formed in the hydrogel. For example, since CNTs tend to aggregate together because of their hydrophobic nature, it is possible that heterogeneous regions may exist in the CP and CNT in the hydrogel of the polymerized procedure [139]. Although CNTs grown in PANi showed high initial conductivity of 2.946 × 10^3^ mS·cm^−1^ [140], the reduced electrical properties of the composite because of agglomeration are still a major issue. In addition, conductive materials, such as CNT and graphene, can cause structural defects when mixed with polymers in the development of conductive hydrogels [125,141].

Limitation of agglomeration in the blending technique leads the in-situ process. This technique improves the interaction between conductive and non-conductive polymers, which provide higher mechanical strength and the highest degree of flexibility of design [126].

#### 2.2.2. In Situ Process

The in-situ growth mechanism was introduced to provide conductivity by polymerizing the conductive component in a nonconductive hydrogel. Since such approach provides the enhanced integration of two components; it is essential to adjust the balance between the material properties, and this requires the process to be optimization for combining the new material properties. For the development of conductive hydrogels growing in situ, some techniques including the growth of metal nanoparticles in bulk hydrogels [142,143], the deposition of CNTs through chemical vapor deposition [113], and the polymerization of conductive polymers have been performed [144].

**Figure 10:**
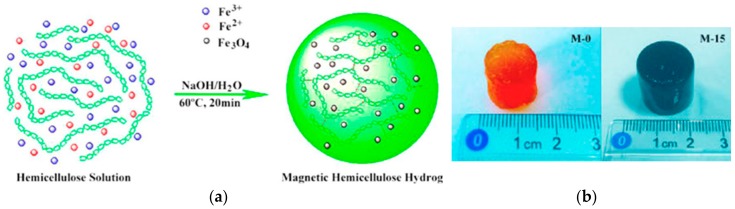
Fe_3_O_4_ nanoparticles synthesized in situ. (**a**) Proposed fabrication of magnetic field-responsive hemicellulose hydrogels in basic media. (**b**) Prepared hemicellulose hydrogel (M-0) and magnetic-responsive hemicellulose hydrogel (M-15) (reproduced from [145] under open access license).

The process of forming an in situ conductive material in a hydrogel is dependent on the type of materials. Metal particles tend to grow into nanoparticles mainly from the form of ions. Before the conductive particles are formed in the hydrogel, the degree of ion dispersion can be an important factor for producing a hydrogel with homogeneous conductivity. Zhao et al. reacted Fe_3_O_4_ nanoparticles preferentially mixed with a hemicellulose solution in the state of Fe^3+^ or Fe^2+^ ions and homogeneously produced with a NaOH solution at 60 °C to form a hydrogel (Figure 10) [145]. The content of the Fe_3_O_4_ nanoparticles controlled the thermal stability, macroscopic structure, swelling behavior, and magnetization of the hydrogel. The in situ preparation of CNTs and polymer composites homogeneously distributes CNTs throughout the hydrogel and increases the weight fraction of the CNTs without impairing the mechanical strength of the hydrogel and enables excellent mixing. For example, the force transfer from the CNTs to the polymer constituting the hydrogel is affected by the homogeneity of the CNTs [141,146,147]. Thus, conductive hydrogels synthesized by in situ techniques can have uniform conductivity, improved reliability, and increased strength of hydrogels.

**Figure 11:**
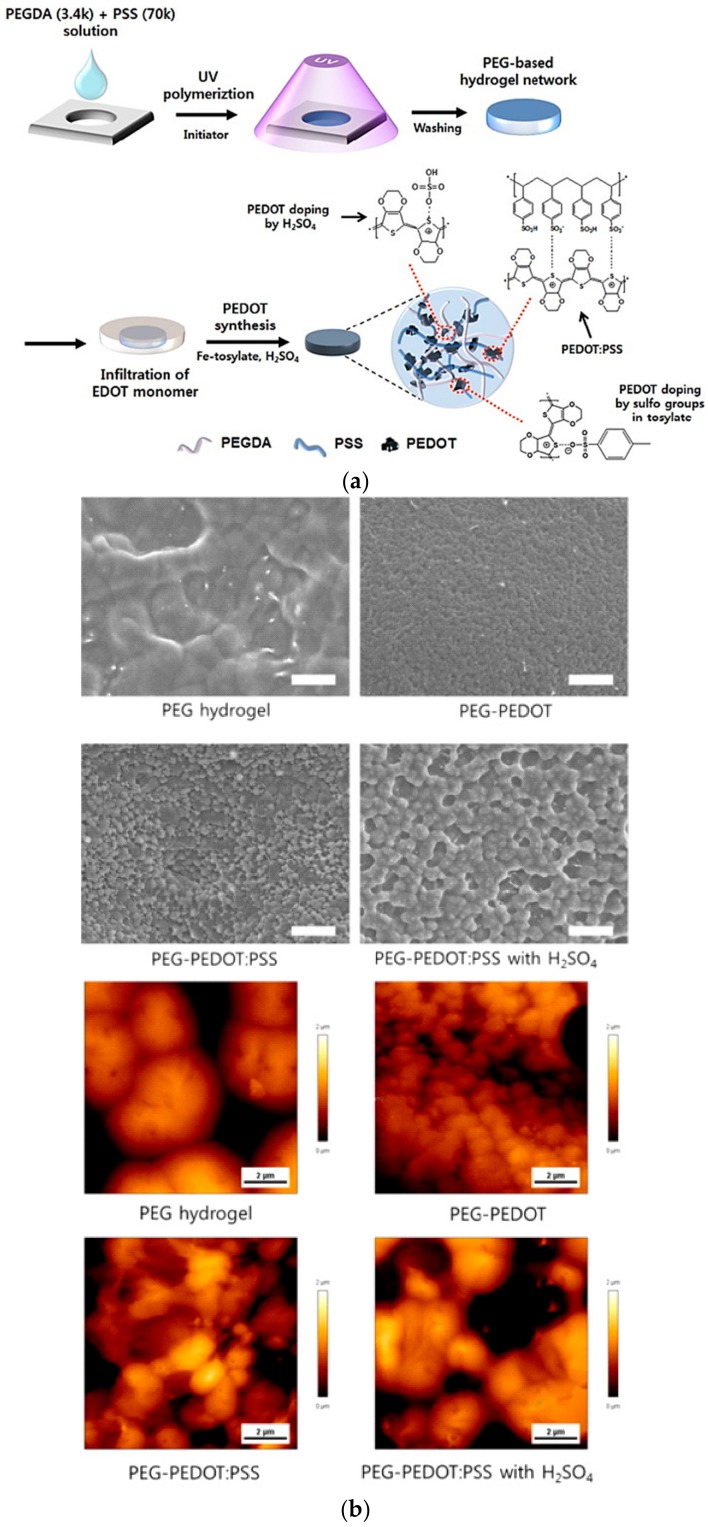
Conductive PEDOT-PEGDA hydrogels using PSS-PEDOT (**a**) scheme of synthesizing conductive hydrogels and (**b**) surface morphology of PEG hydrogel, PEG-PEDOT, PEG-PEDOT:PSS, and PEG-PEDOT:PSS treated with H_2_SO_4_ (reproduced from [148] with permission, copyright Elsevier, 2016).

Incorporating additional substances can further enhance the conductivity in the process of in situ synthesis of conductive hydrogels. Kim et al. synthesized PEDOT-PEGDA hydrogels with high conductivity and moisture contents using PSS-PEDOT (Figure 11) [148]. The incorporation of PSS in a PEG hydrogel promoted the in-situ synthesis of PEDOT in the hydrogel to produce a hydrogel with increase in conductivity that was further enhanced by H_2_SO_4_ treatment.

Nevertheless, in order to grow and synthesize conductive polymers in hydrogels different from blending synthesis, additional techniques and steps may be required to account for the effects of ambient hydrogels. The increase in the synthesis steps can result in reduced reproducibility. Continuous research is necessary to solve the problem of devising a stable material with high reproducibility.

#### 2.2.3. Coating Process

One method that can easily provide conductivity to a hydrogel is to coat the surface of the hydrogel. This method makes it possible to produce electrically conductive hydrogels with appropriate customized physicomechanical properties by utilizing the flexible manufacturing and processing techniques used for the polymers making up the hydrogel. The surface coating is conducted by various chemical reaction methods including click chemistry, reversible split chain transfer, and spinner vision on the surface of the hydrogel material.

**Figure 12:**
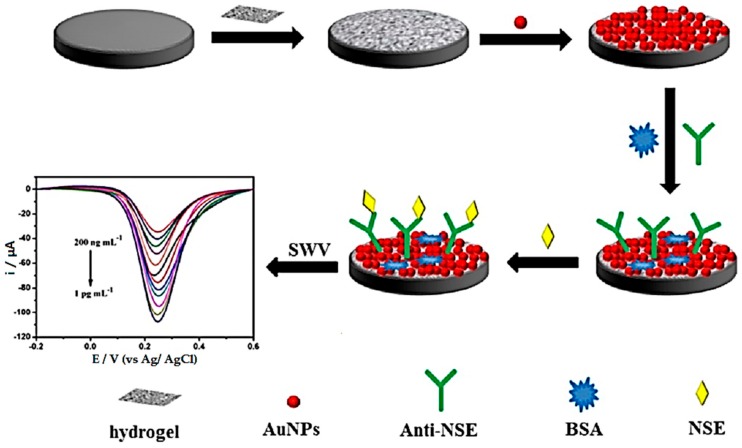
AuNPs electrodeposited conductive hydrogel by adopting a crosslinking method using 1,3,5-benzenetricarboxylic acid as a ligand and Fe3+ as a metal ion was synthesized. The immunosensor of the synthesized conductive hydrogel had a wide linear detection range of 1 pg·mL^−1^ to 200 ng·mL^−1^ and had excellent immunoassay (reproduced from [150] with permission, copyright Elsevier, 2017).

Surface coatings have essential advantages of bioactive interface and drug delivery. Conductive layers bonded to the surface are available for surfaces that are exposed to cells that require biomolecules, such as growth factors and cell adhesion proteins. These are integrated with the materials that make up the biocompatible hydrogel, and diffusion may then be transmitted to cells. Luo et al. developed a method for producing PPy by using controlled nano-porous structures to release controlled dexamethasone in response to an electric current [149]. Wang et al. prepared an electrodeposited AuNP conductive hydrogel by adopting a crosslinking method using 1,3,5-benzenetricarboxylic acid as a ligand and Fe^3+^ as a metal ion (Figure 12) [150].

However, the substances constituting the hydrogel and the components of the coating material act somewhat independently, so it is possible that components with low elasticity may peel off or crack [151]. It is therefore crucial that the polymer coating has sufficient interaction and bonding with the two components at the upper interface to prevent peeling. Xie et al. showed that by forming a hydrogel fiber bundle, it is possible to form a conductive air guiding structure by coating a conductive polymer on its surface [152]. In this experiment, since there was sufficient mechanical bonding between the surface of the fiber nanoscale mat and PPy, peeling off of the coating was not indicated, and the electrochemical properties were similar to those of PPy macromolecules.

Conductive coatings have a variety of advantages associated with the delivery of drugs, including bioactive agents. However, they have limitations in terms of peeling and mechanical differences associated with the interface between the coating and the hydrogel. Therefore, studies should be conducted to overcome this problem.

## 3. Biomedical Applications for Tissue Engineering

It is important that hydrogels used in tissue engineering are provided with additional characteristics such as physical strength or antimicrobial properties, depending on the type of tissue, as well as the conductivity. Conductive hydrogels have been actively studied and utilized in the fields of cardiac, nerve, bone, and skin-tissue engineering (Table 5).

### 3.1. Cardiac Tissue Engineering

Cardiovascular diseases such as myocardial infarction and heart attack occur with abnormal electrical function because of the severe loss of myocardial cells. Compared to other tissues such as bones and skins, the cardiac muscle has a markedly limited regenerative capacity. When myocardial tissue becomes damaged, it forms a fibrotic scar tissue with a permanent loss of myocardial tissue. Many researchers have explored application plans that mimic cardiac tissue [153,154]. Since cardiomyocytes and related progenitor cell populations have been shown to grow exponentially and migrate well by electrophysiological stimulation, conductive hydrogels have been introduced into the applications of tissue engineering to mimic the intrinsic properties of such a cardiac cell environment.

Yang et al. developed a homogeneous electron conducting dual network (HEDN) consisting of a rigid hydrophobic conductive network of chemically crosslinked poly (thiophene-3-acetic acid) and a flexible hydrophobic network of photographic crosslinking methacrylated aminated gelatin [155]. PTAA was synthesized via oxidation-coupling polymerization of 3-thiophene ethylacetate in the presence of FeCl_3_. After the DN hydrogel was formed, the PTAA monomer was introduced into the double network hydrogel by diffusion. Thereafter, a chemically crosslinked PTAA network and a methacrylate aminated gelatin network photocrosslinked to UV were formed at the same time. In this experiment, the Young’s modulus of the HEDN conductive hydrogel was adjustable from 22.7 to 493.1 kPa according to the network ratio. Furthermore, the conductivity had a 10^−1^ mS·cm^−1^, similar to the reported conductivity range of myocardial tissue. Their biological assessment confirmed that brown adipose-derived stem cells survived and proliferated on the HEDN-conducting hydrogel and improved cardiac differentiation efficiency. Jing et al. synthesized a chitosan-dopamine-GO composite conductive hydrogel by oxidizing a mixture of chitosan, GO, and dopamine [156]. In the crosslinking process of dopamine, the chitosan-dopamine-GO crosslinking network was formed. Chitosan-dopamine-GO hydrogel showed three times more adhesive strength than chitosan-dopamine hydrogel. The cell culture results demonstrated that the conductive chitosan-dopamine-GO hydrogel improves cell viability and proliferation of human embryonic stem cell-derived fibroblasts and myocardial cells. Sun et al. revised the CNT/Collagen hydrogel to confirm promoting cell-cell integrity and enhance the functional tissues [157]. In this study, CNTs were added into the collagen solution for gelation. The incorporation of CNTs showed no toxicity and reinforced cell adhesion and elongation of myocardial cells.

In general, composites composed of conductive hydrogels need to promote tissue formation under mechanical stimulation while providing appropriate electrochemical signals in a variety applications of cardia tissue engineering [158]. The design of a conductive hydrogel that mimics natural extracellular matrix (ECM) characteristics requires consideration of both electrical activity and mechanical strength. In particular, the conductive hydrogel studied in cardiac tissue engineering has been focused on improving the elasticity. Hosseinzadeh et al. evaluated polyacrylic acid (PAA)-based conductive hydrogel using aniline polymerization based on Au nanoparticles homogeneously [159]. The Young’s conductive gels were more similar to myocardium, and neonatal rat cardiomyocytes showed an increased expression of connexin 43. Jo et al. synthesized a graphene conductive hydrogel comprised of reduced GO and polyacrylamide (PAAm) [160]. For the preparation of GO-incorporated PAAm conductive hydrogel, the acrylamide/bis-acrylamide solution, the GO solution, and the initiator APS solution were mixed and polymerized at 60 °C. In addition, GO was reduced by treating I-ascorbic acid solution. Reduced hydrogel (r(GO-PAAm)) has an elastic modulus of approximately 50 kPa, which is the same strength as muscle tissue. In addition, an in vitro experiment of C2C12 myoblast showed a significant increase in proliferation and root differentiation compared to a PAAm hydrogel. To provide a mixture of conductivity and bioactivity, Annabi et al. devised a conductive hydrogel integrated with GO nanoparticles and a highly elastic methacryloyl-substituted tropoelastin-based hydrogel [161]. In this experiment, GO nanoparticles imparted conductivity while improving the toughness and elasticity of the treated hydrogel. The improved elasticity of GO particles occurred because of polymer chains and hydrophobicity, hydrogen bonds, and electrostatic interactions between the polymer chains and GO nanoparticles. In addition, the synthesized conductive hydrogel supported active growth and maturation, encouraging the growth and functionality of neonatal rat cardiomyocytes. Many studies have confirmed that conductive hydrogel is biocompatible by successfully transplanting it into rats without causing high inflammatory reactions.

**Figure 13:**
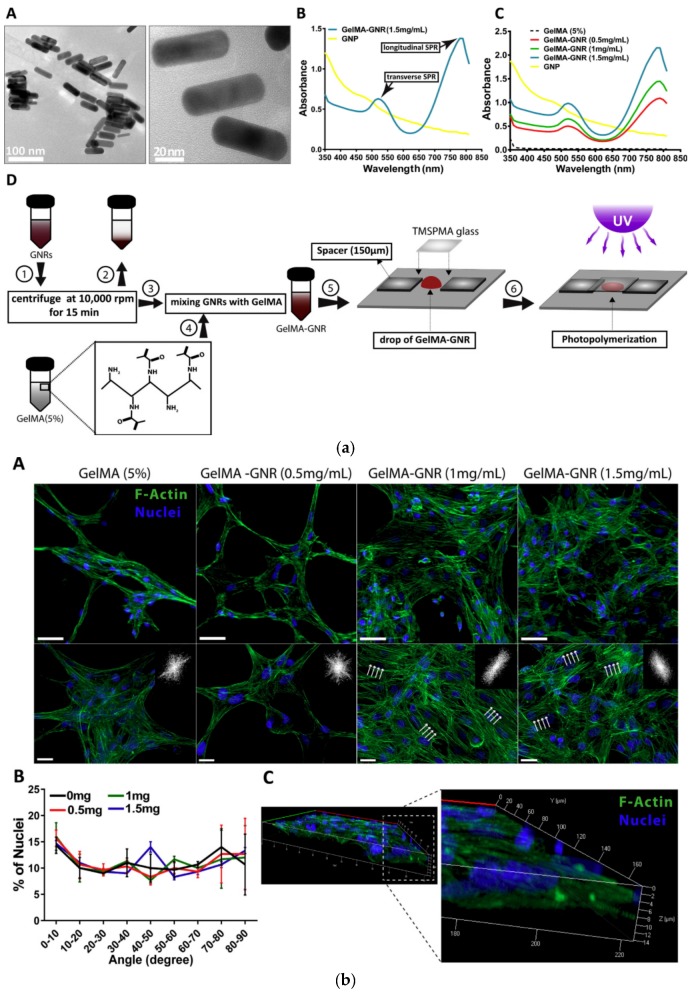
(**a**) Synthesis and characterization of GNR and GNR-GelMA hybrid hydrogels and (**b**) nuclei alignment and F-actin cytoskeleton organization of cardiomyocyte in GelMA only and GelMA-GNR hydrogels (reproduced from [163] with permission, copyright Elsevier, 2016).

Metal nanoparticles have been practically utilized as a conductive hydrogel for cardiac tissue regeneration for improving mechanical properties and biocompatibility. Metal nanoparticles can easily tune the mechanical and electrical properties of a hydrogel depending on their concentration and materials. It is important for myocardial research to synthesize these tunable conductive hydrogels because of the similarity of myocardial cell surroundings. Ahadian et al. devised a conductive GelMA using a palladium-based metallic glass submicron line (PdMGSMW) to increase the mechanical strength [162]. Conductive GelMA-PdMGSMW hydrogel can be varied depending on the concentration of the submicro lines in a hydrogel, which allows for more effective adhesion of C2C12 cells and root canal formation contraction. Navaei et al. developed a GelMA conductive hydrogel containing a UV-crosslinked gold nanorod (GNR) with improved biological and mechanical properties for cardiovascular tissue engineering (Figure 13) [163]. GNR improved the mechanical strength and conductivity of hydrogels. In addition, myocardial cells seeded with GNR-GelMA hydrogel showed excellent cell retention, cell adhesion, and viability. GNR-GelMA also supported myocardial cell beating at concurrent tissue levels. Hosoyama et al. revised collagen-gold biomimetic matrices for cardiac tissue engineering [164]. The collagen-gold conductive hydrogel was prepared by mixing gold nanoparticles in a collagen solution and through thermal crosslinking. The incorporation of gold nanoparticles increased the mechanical strength 5-fold of the conductive hydrogel and improved the healing properties by favoring the migration of progesterone M2 macrophages. Liu et al. developed the PEGylated chitosan hydrogel dispersed with TiO_2_ nanoparticles [165]. To produce spherical TiO_2_ nanoparticles in the hydrogel, TiO_2_ nanoparticles incorporated PEG-chitosan hydrogel was formed by adding Titanium isopropoxide solution to the PEG-chitosan matrix and reacting. The synthesized hydrogel showed improved expansion behavior, and the cell retention activity and adhesion of myocardial cells were improved by the nanoparticle network.

**Figure 14:**
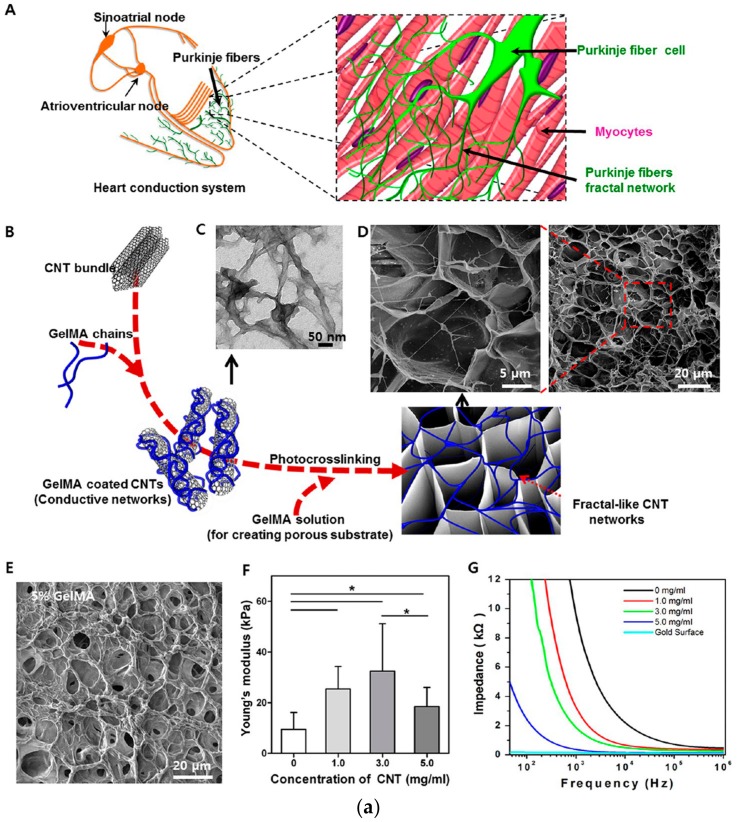

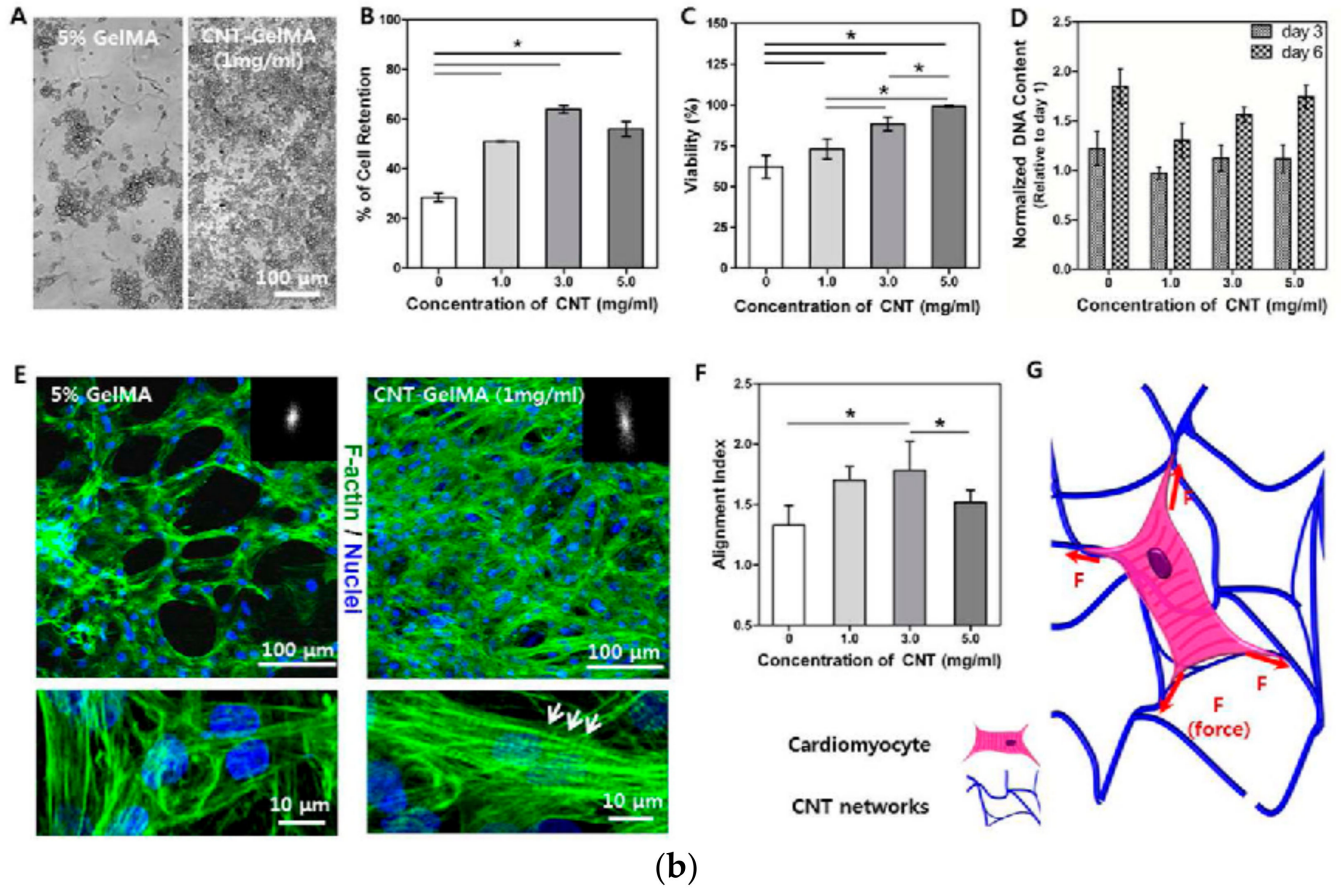
(**a**) Successfully synthesized CNT-GelMA conductive hydrogel and (**b**) improved cardiac cell adhesion and alignment on CNT-GelMA. In this study, CNTs improved cell-cell coupling and cardiac cell adhesion (reproduced from [167] with permission, copyright American Chemical Society, 2013).

CNTs can be aligned in a gelatin methacryloyl (GelMA) hydrogel by using a dielectriophoresis method [162] that allows the hydrogel to provide accurate and adjustable electrical pulse stimulation to cells and tissues. Mouse embryoid bodies were cultured in microwells containing conductive hydrogels with CNTs. This conductive hydrogel enhanced the cardiac differentiation of embryoid bodies when compared to a GelMA only and a random CNT-GelMA hydrogel. Therefore, the conductive hydrogel can provide an electrically efficient and adjustable cell growth platform. In addition, CNTs can be applied to electron-emitting fibrous polymers to improve mechanical strength [166]. Shin et al. synthesized functional cardiac patches by seeding neonatal rat cardiomyocytes on CNT-incorporated photo crosslinkable gelatin methacrylate (GelMA) hydrogels (Figure 14) [167]. In this study, electrically conductive networks within a porous gelatin framework utilized by CNTs showed an improvement in cell-cell coupling and adhesion of cardiac cells. These results proved that the incorporation of CNTs into biomaterials can be exploited to create multifunctional cardiac scaffolds for therapeutic purposes and in vitro studies.

For the study of myocardial tissue, it is necessary to consider a conductive hydrogel that can satisfy both the mechanical strength and the conductivity that mimic the cardiac circumstances and withstand the heartbeat. Conductive hydrogels used in myocardial tissue should be considered for mechanical strength enhancement, such as tensile, and various conductive materials can be incorporated into the hydrogel to meet the need for conductivity.

### 3.2. Nerve Tissue Engineering

Damaged nervous tissue can be treated artificially if the depth of injury is so deep that it is difficult to recover by self-sustenance and will permanently damage a body’s function. Researchers have studied nerve tissue lesions using various strategies. The commercialized treatment method for treating nerve tissue defects is to transplant autografts, allografts, or xenografts to lesions. However, these treatment methods can increase the prevalence of the donor site and evoke an immune-rejection reaction. Therefore, researchers have devised hydrogels that can be used for tissue engineering for nerve tissue regeneration to complement the disadvantages of existing transplantation treatments. Various studies have demonstrated that a conductive environment promotes neuronal proliferation and differentiation by providing an environment around nerve tissue of electrical signal exchange and conduction properties.

**Figure 15:**
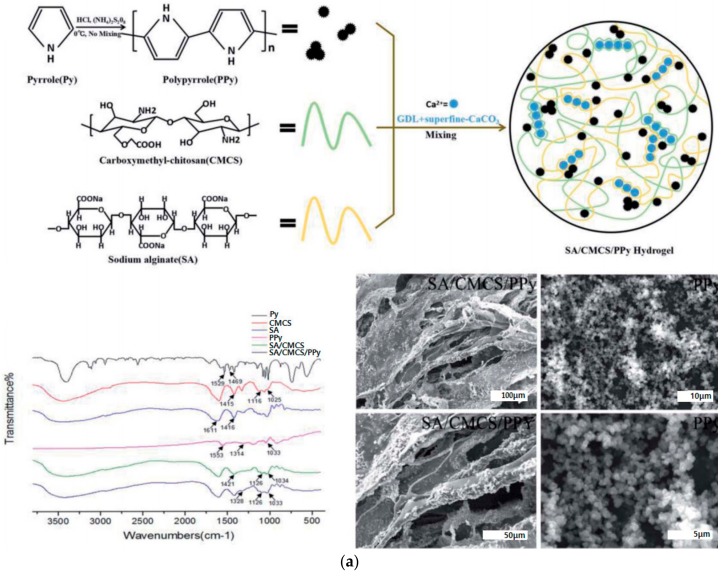

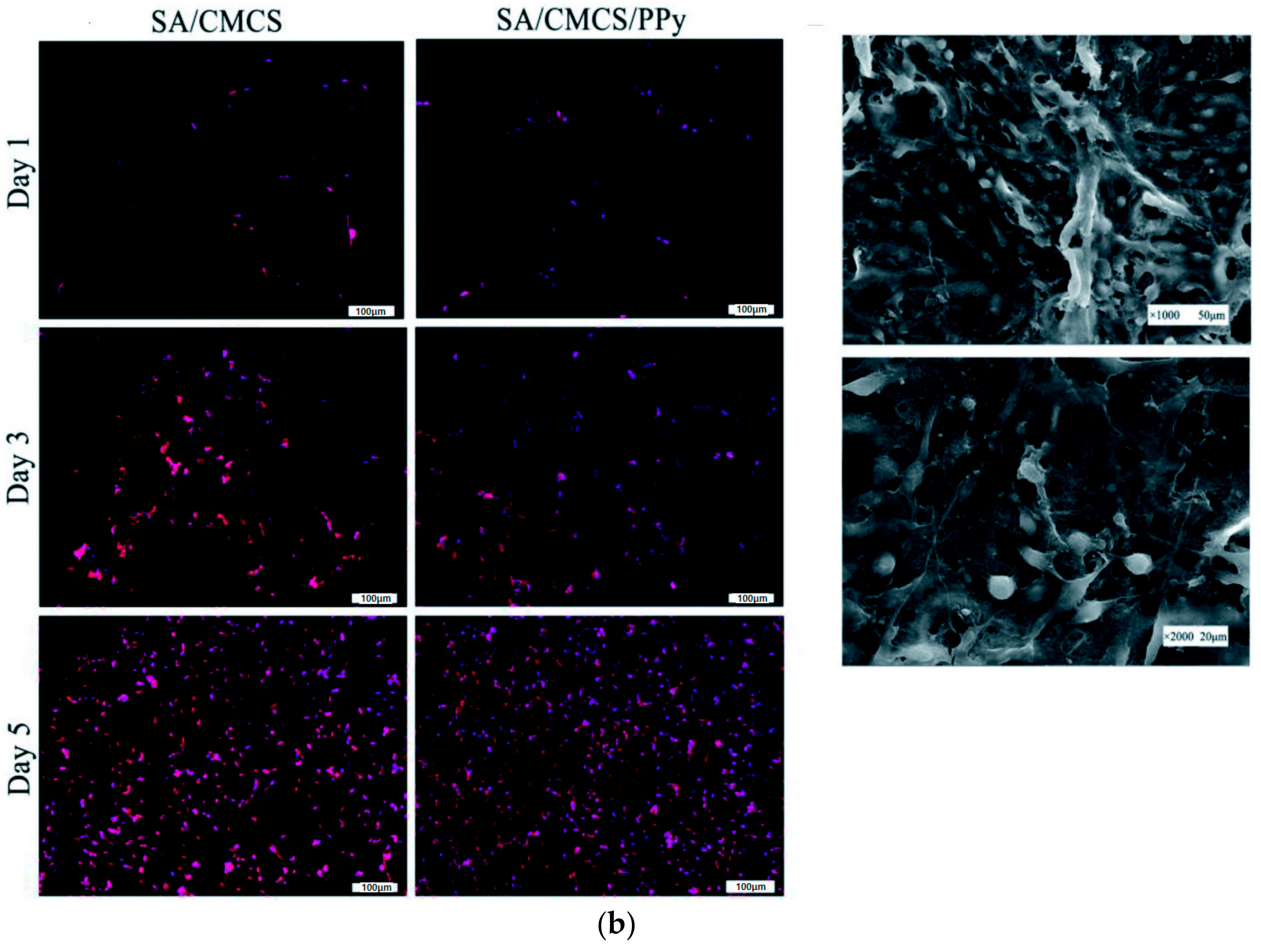
Conductive sodium alginate, PPy, and CMCS hydrogels to aid in peripheral nerve regeneration. (**a**) Sodium alginate/CMCS/PPy hydrogel was successfully synthesized; (**b**) PC12 cells on sodium alginate/CMCS and sodium alginate/CMCS/PPy hydrogel. PC12 cells grew well and adhered to sodium alginate/CMCS/PPy more effectively compared to the control sample (reproduced from [169] under open access license).

In addition, it is essential to test the biocompatibility and conductivity of various conductive hydrogels in nervous tissue engineering applications. Shi et al. prepared an in situ PPy conductive nanoporous cellulose hydrogel [168]. The NCG-PPy conductive hydrogel was prepared by in situ vapor phase polymerization of pyrrole monomers by simply mixing nanoporous cellulose gel (NCG) prepared from aqueous alkali/urea solution with pyrrole monomers in liquid phase and oxidant. The resulting NCG-PPy conductive hydrogel showed a conductivity of 80 mS·cm^−1^. In vitro studies have shown that adhesion of PPy to NCG improved adhesion and proliferation of PC12 cells and showed that the PPy-NCG hydrogel induced neurite outgrowth and had excellent biocompatibility. Bu et al. introduced a method of synthesizing conductive sodium alginate, PPy, and carboxymethyl chitosan (CMCS) polymer hydrogels to aid in peripheral nerve regeneration (Figure 15) [169]. The calcium ion crosslinked sodium alginate/CMCS hydrogels provided by the sustained release system consisting of d-glucono-d-lactone and ultrafiltered calcium carbonate (CaCO3) were coated with PPy particles. The swelling ratio, gelation time, elastic modulus, and porosity of the conductive hydrogel were adjusted according to the content of PPy. The conductivity of the synthesized sample was 2.41 mS·cm^−1^. The prepared conductive hydrogel showed high biocompatibility and cell adhesion and proliferation by culturing PC12, RSC96, and bone marrow-derived mesenchymal stem cells (BMMSCs). In vivo studies confirmed that conductive hydrogel has biocompatibility through subcutaneous inflammatory reactions and can act as a supplement in the nerve conduit. Yang et al. synthesized a conductive PPy/alginate hydrogel by polymerizing PPy chemically in an ionically crosslinked alginate hydrogel [170]. In this study, the alginate hydrogel was immersed in a pyrrole solution to allow the pyrrole monomer to diffuse into the hydrogel. PPy polymerization was initiated by the addition of a chemical oxidant (FeCl_3_) to form PPy within the hydrogel. The cell adhesion and growth of human bone marrow-derived mesenchymal stem cells in PPy/alginate hydrogel were promoted. In addition, the PPy/alginate hydrogels enhanced the expression of neural differentiation markers of human bone marrow-derived mesenchymal stem cells, including Tuj1 and MAP 2 relative to control groups. This study showed that conductive hydrogel can be useful in providing mechanical and electrical signals to stem cells and nerve cells. Imaninezhad et al. prepared MWCNT-PEG conductive hydrogel by mixing polyacrylamide (PA), polyethylene glycol (PEG), and MWCNT [171]. In this study, the nanocomposites composed of 20% *w*/*v* PEG and 0.1% *w*/*v* MWCNT showed the longest neurite outgrowth and average neurite length and increased by 2 and 1.8 times, respectively by electrical stimulation.

Neuronal cells can also induce cell growth and elongation by being provided with adequate mechanical strength. Jafarkhani et al. characterized the mechanical property by synthesizing GO/chitosan hydrogel and analyzed adhesion and proliferation of nerve cells [172]. After mixing the aqueous graphene solution and chitosan powder, lactic acid was added and reacted to produce the GO/chitosan hydrogel. In this study, GO addition induced pore structure and enhanced the mechanical strength of the hydrogel. In addition, the GO/chitosan hydrogel improved growth of nerve cells up to 20%. Zhao et al. developed polyacrylamide/GO/gelatin/sodium alginate hydrogel to enhance peripheral nerve regeneration [173]. The solution of polyacrylamide, GO, gelatin, and sodium alginate was transferred into the mold and heated at 60 °C to synthesize the composite hydrogels. In addition to the physical properties that can be controlled by the amount of GO, the complex hydrogel improved adhesion and proliferation of Schwann cells.

Neurons can induce higher nerve expansion in an environment where orientation is guaranteed. The orientation in the hydrogel can increase the efficiency of nerve regeneration by providing physical cues to neurons. Rose et al. synthesized a matrix by using rod-shaped magnetoceptive microgel to provide structural guidance to neuron cells [174]. The microgels were doped with a small amount of FeO_2_ nanoparticles, allowing alignment to an external magnetic field. In cell experiments, dorsal root ganglions were shown to expand in parallel, demonstrating that hydrogels based on FeO_2_ nanoparticles are capable of forming a variety of microenvironments for neuronal cell growth.

The nerve ECM has various conductivities from peripheral nerve tissues to cerebral cortex tissues [123]. In neural tissue engineering, research has shown the necessity of producing conductive hydrogels that can easily change conductivity corresponding to the different electrical environments of nerve tissues. Xu et al. synthesized a conducting complex nerve conduit with PPy and poly(d,l-lactic acid) (PDLLA) and evaluated its capability to carry the differentiation of rat pheochromocytoma 12 (PC 12) cells in vitro, which determined the ability to encourage nerve regeneration in vivo [175]. After PDLLA, pyrrole, and sodium dodecyl sulfate solution were mixed, FeCl_3_ solution was added to initiate oxidative polymerization to PPy. Depending on the PPy content of the produced nerve conduit, the conductivity was in the range of 15.56–5.65 mS·cm^−1^. PC12 cells were seeded in the conduits and showed an increase in both the neurite-bearing cell proportion and central neurite length. Liu et al. devised an rGOaCNTpega-OPF-MTAC hydrogel with a positive charge and conductivity that passed the positive charge to 2-(methacryloyloxy) ethyltrimethylammonium chloride (MTAC) and chemically crosslinked it to GOa and CNTpega in an oligo (poly(ethylene glycol) fumarate) (OPA) hydrogel [176]. The conductivity of the hydrogel increased step by step during the process of synthesizing the hydrogel. The final conductivity was approximately (5.75 ± 3.23) × 10^−2^ mS·cm^−1^. Biological evaluation also showed a spread of PC12 cells on the conductive hydrogel, which was confirmed by the strong neurite outgrowth of cells on the conductive hydrogel induced during the differentiation process after growth factor treatment. A polyurethane hybrid composite was devised using PSS-doped PEDOT and liquid crystal GO, a polyether-based liner polyurethane and the conductive hydrogel obtained high biocompatibility, conductivity, and flexibility [177]. PEDOT:PSS and polyether hydrogel was synthesized by solution casting formulation. The synthesized polyurethane hybrid composite conductive hydrogel showed 10-times higher conductivity, 1.6-times higher tensile modulus, and 1.56-times the yield strength than a control group. It also supported human neural stem cells growth and the differentiation of neurons. It was confirmed that the produced hydrogel secured biocompatibility, high flexibility, and conductivity.

Conducting hydrogels used in neural tissue studies require physical properties such as aligned morphology and mechanical strength, biocompatibility, and the ability to control the conductivity of the surrounding neural tissues. Conductive hydrogels of neural tissues need to focus on different conductivities depending on the location of various neurological lesions.

### 3.3. Bone Tissue Engineering

Bone tissue engineering undergoes a process initiated by the migration and recruitment of bone origin cells. It is then followed by proliferation, differentiation, and matrix formation [178]. Generally, bone tissue engineering materials require high mechanical strength of osteoconductive characteristics [179]. However, hydrogel has a low mechanical strength and needs to improve its mechanical properties to mimic bone tissue.

The conductive material in a hydrogel should be able to increase the effect of bone conduction and mechanical strength. Gold nanoparticles (GNPs) are known to be the most promising substances for bone tissue regeneration, because they promote osteogenic differentiation of MSCs [180]. Heo et al. synthesized biodegradable hydrogel using GNPs and regenerated bone tissues [181]. The hydrogel contained GNPS in a gel via UV-induced chemical crosslinking using GelMA. The cell experiment showed that the conductive GNP hydrogel significantly increased activity, proliferation, and bone formation, especially in animal experiments. To increase the elastic modulus, roughness, and conductivity, the incorporation of conductive fibers using graphene nanoparticles and PANi into a hydrogel was devised [182]. In cell experiments, the conductive hydrogel-fiber complex retained similar cell adhesion, proliferation, and morphology to human osteoblasts as the non-conductive hydrogel. Ezazi et al. developed a skeletal hydrogel containing hydroxyapaptite, gelatin, and mesoporous silica [183]. This hydrogel was conjugated with PPy macromolecules to confer conductivity, and a model antibiotic (vancomycin) was loaded. The support containing PPy showed superior mechanical properties and a higher proportion of protein than that of the nonconductive support. Even the in vitro experiments confirmed that the osteoblastic cells were contained in gelatin matrix and had survived for 14 days.

Bacterial adhesion to living tissue implanted in the vicinity of bone tissue can disrupt the surface of the graft site and cause infection of the biomaterial. In bone tissue engineering, hydrogels have been studied that can provide antimicrobial properties to prevent bacterial attachment and tissue integration. Ribeiro et al. synthesized the in situ incorporation of local synthetic AgNPs and AuNPs utilizing a tyrosine amino acid in a hydrogel of silk fibroin/nanohydroxyapatite [184]. The synthesized hydrogel proved to be effective for antibacterial activity without damaging the behavior of osteoblasts in AgNPs of 0.5 wt.% or less and AuNPs of all concentrations.

To fabricate a conductive hydrogel to be utilized in bone tissue engineering, it is easy to provide an increase in strength and conductivity by coating the already-hardened hydrogel surface. Pelto et al. demonstrated that PPy-coated PLA scaffolds promote cell growth of adipose-derived stem cells for bone tissue regeneration via physiochemical signaling [185]. A symmetric biphasic pulsed DC voltage of 0.2 V for 4 h at 1 Hz significantly enhanced an adipose-derived stem cell in vitro culture after 14 days. Therefore, the supply of intrinsic conductivity and electrical stimulation of a CNT material provides an overall effect that promotes the osteogenic differentiation of stem cells and regulates the activity of cells indispensable for the regeneration of bone tissue.

**Figure 16:**
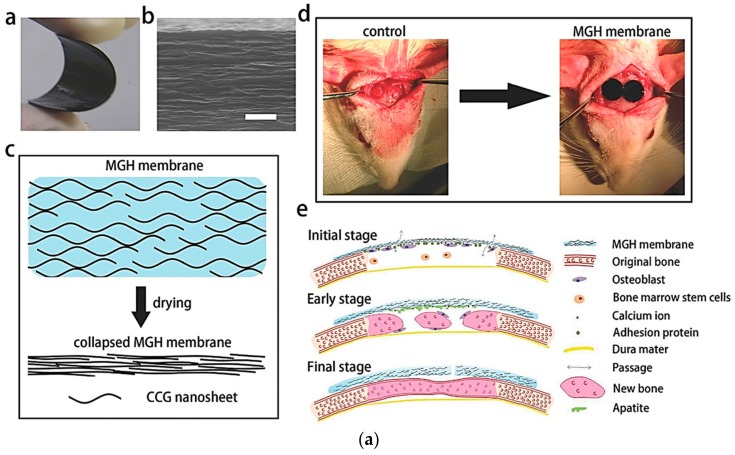

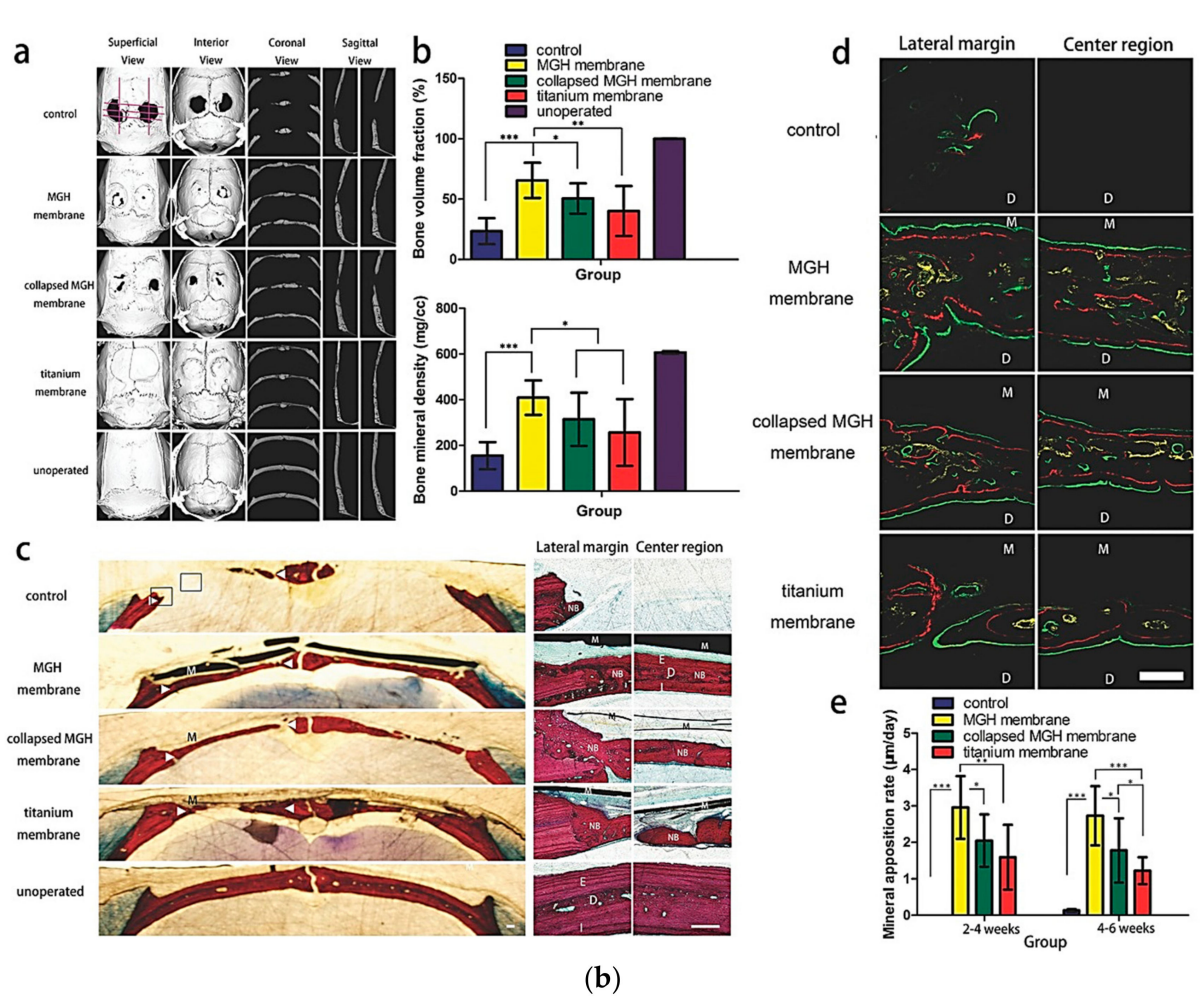
(**a**) Multilayered graphene hydrogel reference for bone regeneration. (**b**) Graphene hydrogel improved the physical properties of mechanical strength and flexibility, and showed improved adhesion on osteoblast and bone tissues (reproduced from [186] with permission, copyright Wiley, 2016).

However, to provide conductivity for regenerating bone tissue, research has been conducted to simultaneously improve mechanical strength and conductivity of biodegradable hydrogels using conductive materials. Lu et al. synthesized a multilayered graphene hydrogel as a reference to utilize in bone regeneration (Figure 16) [186]. It was proved that the chemically synthesized, graphene-based hydrogel properly maintained osseous space and promoted early osteogenesis. In addition, the graphene hydrogel improved the mechanical strength, flexibility, and adhesion of osteoblast and bone tissues. Chen et al. developed a conductive nano-PLA scaffold with well-dispersed PANi nanostructures that promoted osteogenic differentiation and combined the properties of 3D matrices [187]. The scaffold structure and content of polyaniline nanoparticles formed in situ were confirmed, and bone MSCs derived after three weeks were cultured on a composite support. As a result, it was confirmed that expression levels of alkaline phosphatase, osteocalcin, and runt-related transcription factors of bone MSCs on the composite support increased.

As mentioned above, the conductive hydrogel in the osteocyte study investigated the porous structure by securing adequate conductivity and providing the same environment as osteocytes. This could be a key factor of research on conductive hydrogels to significantly promote osteogenic differentiation by providing an environment of structural conductivity.

### 3.4. Skin Tissue Engineering

Skin is the largest organ of the human body that protects the internal environment from the outer environment. Artificial skin regeneration is an emerging substitute to traditional wound healing strategies. Conducting polymers have been reported to support the growth of fibroblasts and keratinocytes [188]. In addition, their antibacterial properties [189,190] make them a promising biomaterial for wound healing. The CSH hydrogel synthesized by free radical polymerization of AA and conductive polymer (Dch-PPy) in presence of FeCl_3_ mimic human skin [191]. The reversible ionic interaction between carboxylic groups of PAA and NH group of PPY and ferric ion leads to a double network hydrogel with mechanical and electrical self-healing properties and ultrastretchability (1500%). Deng et al. prepared hybrid cryogel of PPy, and PANi exhibited photothermal property, elasticity shape memory, and cytocompatibility to the L929 cell [192]. Another injectable conductive hydrogel prepared by chitosan-g-polyaniline and poly (ethylene glycol-co-poly (glycerol sebacate) (PEGS-FA) exibited electroactivity, biocompatibility, and antibacterial activity. The hydrogel with 1.5 wt.% crosslinker showed excellent in vivo clotting, collagen deposition by upregulating the expression of wound healing growth factors such as VEGF, EGF, and TGFβ [193].

## 4. Conclusions and Future Perspectives

This review focused on performing a variety of assessments of conductive materials, manufacturing methods of the conductive hydrogel, and applications for the biomedical area based on studies reported in various papers. Most initial studies on conductive hydrogels focused on evaluating whether conductive materials can be adequately used in the biomedical field. Conductive materials with low mechanical properties, low processability, and bad biocompatibility that are not compatible in vivo were adopted for use in hydrogel manufacturing technology using existing verified materials, resulting in the synthesis of a conductive hydrogel that simultaneously possessed the strength of a hydrogel and conductivity. The importance of an electrically conductive material combined with a proper blending technique and manufacturing method is the key to developing a useful composite hydrogel suitable for applications in the biomedical field. Although this approach can solve the processability and mechanical properties of reduced electrical conductivity and interactions between hydrogels and conducting polymers, the application range of these hydrogels is restricted.

In many reported studies, the biocompatibility and biodegradability testing of conductive hydrogels has been limited to in vitro screening. It is necessary to develop a material constituting conductive hydrogels so that it can be applied to actual patients through proper functional animal research before being used in the field of clinical applications. It is apparent that this is a promising application field, since conductive hydrogels synthesized via a conductive material can be provided to tissue that requires electrical stimulation in the body such as nerve detection and stimulation, and to the regeneration of muscular cells; they can also be used as a biological electrodes in the body.

Artificial skin is an alternative for autologous skin grafting and wound healing. Self-healing and electrical conductivity are the two most crucial properties in the advancement of artificial skin. Conducting polymers with high conductivity and stretchable properties may hasten the development of skin substitution. Moreover, their antibacterial properties make them suitable for wound healing. Conductive polymers in 3D printing may take tissue engineering applications to the next level in the near future. This method may enhance processability, mechanical properties, biocompatibility, and tissue function. Carbon-based nanomaterials play an important role in the generation of conductive polymer, which is expected to stimulate the growth and activity of the electrically excitable cells. Subsequently, coupling of graphene in 3D printing technology has extended their applications.

However, many technical challenges have yet to be solved in this field, and many opportunities are available for researchers to develop hydrogels with strength and conductivity that are suitable for use.

## Figures and Tables

**Table 1:** Properties of Metal Nanoparticles.

Kinds of Nanoparticles	Size (nm)	Shape	Advantages	Disadvantages	Application
Gold Nanoparticles (AuNPs)	1–60	Spherical Rod Polygonal Floral	High stability Low cytotoxicity in initial step Possibility of high scale production	Relatively weak optical signal Long-term cytotoxicity High price	Labelling and visualization, diagnostics, therapeutics, catalysis, cancer cell treatment
Silver Nanoparticles (AgNPs)	4–120	Spherical Wire Oval Polygonal Rod	Anti-microbacterial High optical signal	Cytotoxicity Low stability before surface treatment High price	Anti-microbial, gas/vapor sensing, water sterilization, cancer cell treatment
Platinum Nanoparticles (PtNPs)	10–100	Spherical Cuboidal Floral	Catalysis High optical-signal High stability	High price Cytotoxicity	Biosensing of molecules, enhancement of bone strength, detection of cancer cells
Iron Oxide Nanoparticles	4–45	Tube Spherical Cluster	Super-paramagnetic property Low cytotoxicity Economical	Weak strength Low stability Toxic solvent is needed	Gas sensing, magnetic resonance imaging
Zinc Oxide Nanoparticles	20–600	Flower Rod Wire Sheet	Piezo- and pyroelectric Wide range of UV absorption High optical signal Economical Anti-bacterial effect	Cytotoxicity Low stability Toxic solvent is needed	Photocatalyst, absorber of UV radiation, biosensors, gas sensing

**Table 2:** Bulk Properties of Conductive Polymers.

Kinds of Conductive Polymers	Conductivity (mS·cm^−1^)	Advantages	Disadvantages	Application
Polypyrrole (PPy)	10^3^~5 × 10^4^	High conductivity High stability Biocompatibility High mechanical strength	Easy to Fragile Susceptible to irreversible oxidation Insoluble in water	Biosensors, antioxidants, drug delivery, neural prosthetics, tissue engineering
Polyaniline (PANi)	10^2^~10^8^	High conductivity High stability High conductivity Water solubility	Lack of plasticity Poor cell adhesion and growth Low solubility	Biosensors, antioxidants, drug delivery, bioactuators, food industry, tissue engineering
Polythiophene (PT)	10^−1^~10^−4^	Good optical property Biocompatibility Can obtain various functions according to the reactions	Low conductivity Low stability Low solubility	Biosensors, food industry, tissue engineering
Poly (3,4-ethylene dioxythiophene) (PEDOT)	3 × 10^5^~5 × 10^5^	High stability High conductivity Biocompatibility High mechanical strength Water solubility (doped with PSS)	Relatively low mechanical strength	Antioxidants, drug delivery, neural prosthetics electrode
Poly(p-phenylene vinylene) (PPV)	1~1 × 10^5^	Its precursors can be manipulated in aqueous solution Good optical properties High stability	Insoluble in water Doping is essential to increase conductivity	Biosensors light-emitting diodes Photovoltaic devices

**Table 3:** Bulk Properties of Carbon Materials.

Kinds of Carbons	Conductivity (mS·cm^−1^)	Advantages	Disadvantages
Graphene	10^8^~10^9^	High mechanical strength High conductivity Easy synthesis	Oxidative stress Serious aggregation Toxicity Hydrophobicity
Graphene Oxide	Depend on oxidation and humidity (10^−1^~10^−5^)	Biocompatibility Hydrophilicity Interacting with various inorganic and organic materials Controllable electrical/optical properties	Low conductivity (or even insulator) Sensitive to humidity Weak mechanical strength
Carbon Nanotube (CNTs)	10^7^~10^8^	High mechanical strength High conductivity Magnetic property	Oxidative stress Toxicity Hydrophobicity Additional synthesis step

**Table 4:** Technique of Conductive Hydrogels.

Methods	Advantages	Disadvantages
Blending synthesis	Easy and simple process No additional techniques High reproducibility High stability of conductivity	Low conductivity of hydrogel Weaken hydrogel mechanical strength Difficulty of gelation Heterogeneous conductivity
In situ synthesis	Homogeneous conductivity in hydrogel Enhance hydrogel strength Uniform processability High conductivity of hydrogel High stability of conductivity	Additional techniques are needed Additional step can be needed Low reproducibility
Coating process	Simple process Giving conductivity easily in various shapes of hydrogel	Potential for coating damage Low stability of conductivity Heterogeneous conductivity

**Table 5:** References of Tissue-Engineering Applications.

Application Category	Synthesizing Method	Conductive Materials	References
Cardiac Tissue Engineering	In situ Process	poly (thiophene-3-acetic acid), TiO2NPs, CNTs	[155,162,165,167]
	Blending Process	AuNPs, rGO-PAAm, GO, CNTs, GNR	[153,157,159,160,161,163,164]
Nerve Tissue Engineering	In situ Process	PPy, rGO-CNTs	[168,170,175,176]
	Coating Process	PPy	[169]
	Blending Process	CNTs, GO, FeO2, PSS-PEDOT, GO	[171,172,173,174,177]
Bone Tissue Engineering	In situ Process	AuNPs, AgNPs, PANi	[184,187]
	Coating Process	PPy	[185]
	Blending Process	GNPs, Graphene, PANi, PPy, Graphene	[181,182,183,186]
Skin Tissue Engineering	Blending Process	PPy, PANi	[191,193]
	In situ Process	PANi, PPy	[192]
